# Our New York Letter

**Published:** 1894-06

**Authors:** Wm. L. Russell

**Affiliations:** 101 West 95th Street; New York


### CORRESPONDENCE.

### OUR NEW YORK LETTER.

New York, May 15, 1894.

Quite a number of useful, practical points were brought out at the last meeting of the Section in Pediatrics in a discussion relating principally to the nursing period of infants. The first paper was read by Dr. E. A. Tucker, resident physician of the Sloan Maternity Hospital, and had reference to the management of the breasts and nipples before and during the puerperal period. He said that in many cases no previous preparation was necessary, and the use of a firm binder and perhaps gentle massage for the relief of pain were the only measures required during the puerperal period. Careful cleanliness of the nipples should be observed during pregnancy, as they were liable to become crusted, but no astringent should be used, and manipulation should be avoided unless indicated for the correction of an imperfection.

During the period of lactation, the care of the child's mouth became an important factor in the management of the nipples. A cleansing solution should be used in the child's mouth and on the nipple both before and after nursing. A saturated solution of boric acid was very satisfactory. The nipples should also be kept perfectly dry, and to this end a powder of bismuth subnitrate and salicylic acid could be sprinkled on them. The child should not be allowed to hold the nipple in the mouth except when actually nursing, and although it was not the popular impression, he had found it quite possible to train babies to sleep all night almost from birth. For tender nipples, silver nitrate solution or fifty per cent. alcohol could be applied, or cocaine could be used before nursing, but it was necessary to wash it off carefully before the child tasted it. Cracking of the nipple occurred at the summit or at the base, the latter being the more troublesome location. The application of an eight per cent. silver nitrate solution, with careful drying, and the use of the powder were the most useful measures of treatment. A nipple shield must then be used. That known as the Barcley was the best he had found, but the rubber part he replaced by a large white bottle nipple, which could be inverted and cleaned. One to three applications of the silver nitrate were usually sufficient. Caking of the breast might be relieved by gentle stroking toward the nipple *without* expressing any milk. This treatment caused relief for several hours, and was better than the extracting of milk. Free catharsis with salines and the use of the binder could be added.

Mastitis was invariably the result of infection through the nipple, and was ushered in by chill, fever, and pain. Drainage of the breast could be effected by nursing, after which a cold coil or an ice-bag should be applied, salines being also administered. If treatment were not begun early, abscess would result. Should this be inevitable, it should be encouraged by poulticing and treated by incision.

When it became necessary for any reason to dry up the milk, the very cessation of nursing, with the use of a firm binder, was usually sufficient. Salines might also be used, but belladonna and other agents recommended were superfluous. Dr. H. Fruitnight said that exposure of the nipple to the air daily during the later months of pregnancy was a useful measure in hardening them. He did not think belladonna necessary to dry up the milk. Dr. Floyd Crandall believed the use of astringents for hardening the nipples was a pernicious practice which should be discouraged.

### DIET OF NURSING WOMEN.

A paper on this subject was read at the same meeting by Dr. Jerome Walker, of Brooklyn. He said that a varied diet was needed as a rule. To this might be added beef peptonoids and malted milk in special cases. Malt liquors should rarely be used, and then only moderately. Extract of malt was much better, and a glass of milk with ginger in it taken at night was of some value. Relief of constipation was highly important, but mild laxatives only should be used. The child should be nursed at regular intervals, and rest for the mother should be secured by having some one take care of the baby occasionally. Outdoor air, a contented mind, and good food well cooked were of the greatest importance. If the milk was scanty, milk should enter largely into the mother's dietary.

Dr. Tucker said that it was customary at the Sloan Maternity to restrict the patient to milk during the first two days after confinement. To this was added on the third day oatmeal, soup, vegetables and sauces, with bread and butter and tea. On the fifth or sixth day the regular diet was given. If the milk failed after the third day, the patient was given milk between her meals, about three pints daily.

Dr. Fruitnight thought the use of malt liquors by nursing women was to be deprecated. Dr. Winter had found that many nursing women took tea to excess, and that this was sometimes the cause of a sleepless, colicky baby. Dr. S. Baruch attached great importance to emotional influences in nursing women. He had used metrolastis, castor oil leaves and various other agents said to stimulate the flow of milk, but had found them useless.

### INDICATIONS FOR WEANING.

This was the subject of a third paper read by Dr. L. Emmett Holt. Mothers were sometimes physically unable to nurse, and when this was evident he thought it unwise to persist, as artificial feeding was more successful when begun at birth. The test of successful nursing was to be found in the steady growth of the infant. The weight must increase steadily, and the scales should be used twice weekly. If the weight decreases some fault in the nourishment was surely present. Disturbed sleep, fretfulness and crying, irregular, unhealthy stools, sometimes of a green color, were other indications of the same import. When the milk was scanty, prolonged nursing might be noticed. The infant might hold the nipple for half or three-quarters of an hour, or it might seize it with avidity, only to drop it in disgust in few minutes.

Where there were any grounds for suspicion, an examination of the milk should be made. This should embrace the following points:

1. *Quantity.* If the breasts were full and hard at nursing time nothing was to be feared in that score. In other cases the only resource was to weigh the child before and after nursing, scales registering half ounces being used.

2. *Reaction,* which should always be alkaline.

3. *Specific Gravity,* the normal being 1020 to 1035.

4. *Microscope;* to detect the presence of colostrum, blood, or pus corpuscles.

5. *The amount of fat,* this being determined by a Marchand's tube, or a Babcock centrifugal machine.

The most satisfactory examination was that of a specimen taken from the whole quantity obtained by milking with a breast pump, as that first drawn contained more protein than the average, while the last contained more fat. When the diet was overstimulating and overabundant the milk might be found very large in quantity and overrich. The treatment for such a condition was simply to stop all alcohol, and reduce the quantity of meat, at the same time requiring the mother or wet-nurse to take outdoor exercise. Scanty and poor milk was found in the delicate and anemic. The specific gravity might be but 1024 to 1027, and the percentage of fat not more than two or three. In such a case nursing had better be stopped immediately. If four per cent. of fat be present, however, an attempt might be made to improve the condition by means of fresh air and good food, with iron if anemia existed. Malt liquors in moderation, and better, malt extract, were of some value. It was important too that rest at night be secured. If the mother were very nervous, the prognosis was not very good.

Diminution in the quantity of milk without change in the quality was seldom observed. The best means of overcoming such a condition was to add milk to the dietary. It was more common to find a large quantity of milk of poor quality. The breasts might be found full and overflowing, but the specific gravity would be low and the percentage of fat low. Cessation of nursing was almost invariably the outcome of such cases.

### OPIUM FOR EPILEPSY.

At a recent meeting of the Academy Dr. Jos. Collins read a paper in which he set forth his experiences with this agent. He stated that it had been suggested about a year ago, and that he had tried it on fifty patients. The method of treatment was to give from half a grain to two grains daily, and gradually increase to fifteen grains. After a few weeks the opium was discontinued suddenly and large doses of bromide (half a drachm four times daily) were given for a short time, and then reduced. In twenty cases which had been observed closely, all except one had been benefited, and in two there had been no return of the fits thus far. He did not regard the drug as a specific, or as uniting with the bromides, but he thought it a valuable adjuvant, and one that would take a permanent place in the management of this obstinate disease.

WM. L. RUSSELL.

101 *West 95th Street.*

---

### RULES AS TO TIME OF RUPTURING THE AMNIOTIC SAC IN LABOR.

1. In multipara, rupture when os is fully dilated.

2. In primipara, delay until the soft parts are also dilated.

3. In cases of face and breech presentation, delay in rupturing the sac is best.

4. Where the pelvis is small and the fetus large, delay rupturing.

5. In premature labor, with a dead fetus, rupture early.

6. Rupture the sac early when the membranes are unusually thick, tough and unyielding.

7. When speedy delivery is demanded, rupture early and dilate with the fingers.

8. Rupture the sac when an excessive amount of amniotic fluid retards labor.

9. When version is necessary, and can be accomplished by bimanual manipulation, perform this operation before rupturing.

10. Remember that a dry labor is always to be deprecated, hence do not rupture at all, unless for good reasons and the case demands it.

W. B. CONWAY, M.D.,
H. M. EDWARDS, M.D., Ph.D.

510 *Barber Street, Athens, Ga.*

